# Characterizing insomnia and sleep quality in a cohort with tension type headache

**DOI:** 10.1515/med-2026-1500

**Published:** 2026-09-24

**Authors:** Elena Korabelnikova, Vasilii Tkachenko, Alexey Danilov, Anastasiia Badaeva, Luay Rashan, Sergio Modafferi, Damiano Galimberti, Andrea Cavallaro, Ekaterina Vitish, Anastasiia Vitish, Viacheslav Novikov, Alena Sidenkova, Vittorio Calabrese

**Affiliations:** Department for Nervous Diseases, I.M. Sechenov First Moscow State Medical University of the Ministry of Health of the Russian Federation, Moscow, Russia; Loginov Moscow Clinical Scientific Center, Moscow, Russia; Department for Pathological Physiology, I.M. Sechenov First Moscow State Medical University of the Ministry of Health of the Russian Federation, Moscow, Russia; Biodiversity Unit, Dhofar University, Salalah, Oman; University of Catania, Department of Biomedical and Biotechnological Sciences, Catania, Italy; International Longevity Science Association (ILSA), Milan, Italy; University of Catania, Department of General Surgery and Medical-Surgical Specialties, Catania, Italy; Ural State Medical University, Ekaterinburg, Russia

**Keywords:** chronic tension type headache, insomnia disorders, polysomnography

## Abstract

**Objectives:**

Emerging literature data indicate insomnia as an risk factor for headache or migraine onset. It is generally recognized that headache and insomnia represent very common and major complaints worldwide, while finding exisit that indicate a symmetrical association, with headache being more associated with insomnia than the other way around. Therefore, in the association of headache and insomnia, other factors associated with headache or migraine probably play a role, as well. The aim of the study was to evaluate the clinical and pathogenetic relationships between insomnia and chronic tension-type headache (CTTH).

**Methods:**

A prospective cohort study included 65 patients with CTTH and comorbid chronic insomnia. Participants underwent clinical evaluation, assessment of headache severity (VAS, HIT-6), insomnia (PSQI, ISI), emotional state (Beck, Sheehan scales), and quality of life (SF-12). A subset (n=17) underwent polysomnography (PSG).

**Results:**

All patients reported poor sleep quality. A significant discrepancy was found between subjective sleep perception and objective PSG data (p<0.001). Patients with more severe insomnia had significantly higher levels of anxiety (p<0.001), depression (p=0.025), central sensitization (p<0.001), and lower quality of life (p<0.002). Correlation analysis revealed that worse sleep parameters (e.g., reduced REM, increased wakefulness) were associated with higher headache impact, emotional distress, and central sensitization, particularly in the mild insomnia group.

**Conclusions:**

Insomnia in CTTH patients exacerbates the condition not primarily by intensifying pain itself, but by amplifying its negative impact on emotional state and quality of life, and by strengthening the interconnections between various clinical and psychophysiological manifestations. Treating insomnia is crucial for improving overall patient well-being and pain tolerance.

## Introduction

Tension-type headache (TTH) is a primary headache disorder characterized by bilateral, mild-to-moderate pain, often described as pressure or tightening, and not exacerbated by routine physical activity. Lifetime prevalence estimates range from 20 to 87 %, with peak incidence in women aged 35–39 years [1], 2]. According to the Global Burden of Disease 2016 study, approximately 3 billion people worldwide suffer from headache disorders, with TTH affecting nearly 1.9 billion individuals [3]. Chronic tension-type headache (CTTH) significantly impairs quality of life and poses substantial social and economic burdens [4].

Sleep disturbances, anxiety, and depression are frequent comorbidities in CTTH. Insomnia prevalence in CTTH is 1.8 times higher than in the general population [5], 6], with 68–84 % of patients reporting daily sleep impairment and 26–72 % identifying sleep deficiency as a trigger for headache attacks [7]. Anxiety and depressive disorders are observed in 21.4 and 14.2 % of patients, respectively [8]. The relationship between CTTH and insomnia is likely bidirectional, mediated by shared neuroanatomical, genetic, chronobiological, and neurochemical pathways [9], [10], [11]. Evidence suggests that sleep disturbances can exacerbate headache severity and facilitate chronification, while chronic pain itself may disrupt sleep.

Accumulating evidence indicates that mitochondrial dysfunction driven by sleep deprivation may represent a key convergent biological mechanism linking insomnia and CTTH [12]. Sleep loss disrupts oxidative phosphorylation, reduces cytochrome *c* oxidase activity, decreases mitochondrial membrane potential, and impairs ATP production, leading to excessive generation of reactive oxygen species and oxidative stress [13]. Alterations in Keap1/Nrf2 signaling and activation of PINK1/Parkin-mediated mitophagy reflect impaired mitochondrial quality control and reduced neuroprotective capacity in insomnia. Sleep deprivation alters the homeostasis of peripheral tissues, and insomnia is associated with peripheral inflammation and immune dysfunction. These bioenergetic disturbances contribute to neuroinflammation, central sensitization, and impaired neuronal resilience, thereby promoting headache chronification and sustaining the insomnia–pain “vicious cycle.”

The pathophysiology of CTTH involves both peripheral and central mechanisms, including sustained pericranial muscle tension, myofascial trigger points, peripheral nociceptor sensitization, and central sensitization of spinal and supraspinal neurons, as well as neurotransmitter imbalance, purinergic and nitric oxide dysregulation, and oxidative stress [14].

The convergence of these mechanisms with sleep deprivation–induced mitochondrial dysfunction may explain the high comorbidity between CTTH and insomnia and their mutual reinforcement, influencing affective and cognitive functioning and maintaining long-term tension pain.

This study aimed to evaluate the clinical and pathogenetic relationships between insomnia and chronic tension-type headache.

## Materials and methods

A prospective cohort study was conducted involving 65 patients with chronic tension-type headache (CTTH) and comorbid chronic insomnia. The cohort consisted of 54 (83.1 %) women and 11 (16.9 %) men. The median age was 43 [15], [16], [17], [18], [19], [20], [21], [22], [23], [24], [25], [26], [27], [28], [29], [30], [31], [32], [33], [34], [35], [36], [37], [38], [39], [40], [41], [42], [43], [44], [45], [46], [47], [48], [49], [50], [51], [52], [53], [54], [55], [56], [57], [58], [59], [60] years.

Inclusion criteria: patients with CTTH meeting the diagnostic criteria for CTTH according to the International Classification of Headache Disorders, 3rd edition (ICHD-3), and comorbid chronic insomnia, as per the Diagnostic and Statistical Manual of Mental Disorders, 5th Edition (DSM-5). Exclusion criteria: absence of a signed voluntary informed consent form to participate in the study.

All patients met the criteria for chronic headache and chronic insomnia. A long-term history (over 6 years) of insomnia was noted in 26 (40 %) patients, and of headache in 48 (73.8 %) patients.

Patients underwent clinical examination, assessment of headache severity, and objective and subjective evaluation of the severity and nature of insomnia, quality of life, and emotional state.

Headache severity and characteristics were assessed using the Visual Analogue Scale (VAS) for pain (0 - no headache, 10 - the most severe pain), the Pain Catastrophizing Scale, the Central Sensitization Inventory (CSI), and the Headache Impact Test (HIT-6). To investigate the severity of insomnia, the following were used: the VAS for sleep (1 - best sleep quality, 10 - worst sleep quality), the Pittsburgh Sleep Quality Index (PSQI), and the Morin Insomnia Severity Index. The Sheehan Anxiety Scale and the Beck Depression Inventory (BDI) were used to assess emotional state. Quality of life was assessed using the Short Form Health Survey (SF-12) and a VAS for health (0 - worst health state, 100 - best health state).

A subset of patients (n=17) underwent polysomnography using a WeinmannSomnolab (PSG) device, which assessed parameters such as total sleep time (TST), and the duration of sleep stages: stages 1 and 2 (S1, S2), the rapid eye movement (REM) phase, S3 and S4 (delta sleep), and wake after sleep onset (WASO).

All patients provided signed voluntary informed consent to participate in the study.

### Statistical analysis

Statistical analysis was performed using the StatTech v.4.0.7 program. Descriptive statistics are presented as median and interquartile range – Me [Q1-Q3]. Quantitative indicators were assessed for normality of distribution using the Shapiro-Wilk test and the Kolmogorov-Smirnov test.

Comparison of two groups on a quantitative indicator with a normal distribution, assuming equal variances, was performed using Student’s *t*-test; if variances were unequal, Welch’s t-test was used. Comparison of two groups on a quantitative indicator with a distribution that deviated from normal was performed using the Mann-Whitney *U* test.

The direction and strength of the correlation between two quantitative indicators were assessed using Pearson’s correlation coefficient (for normally distributed compared indicators) and Spearman’s rank correlation coefficient (for indicators with a non-normal distribution); the strength of the relationship was interpreted using the Chaddock scale.

Differences were considered statistically significant when the observed value of the two-sided test was less than the significance level of 0.05 (p<0.05).

The study was conducted in accordance with the principles of the Declaration of Helsinki and was approved by the Local Ethics Committee of the Federal State Autonomous Educational Institution of Higher Education *I.M. Sechenov First Moscow State Medical University of the Ministry of Health of the Russian Federation (Sechenov University)* (Protocol No. 14–25, approved on June 19, 2025). Written informed consent was obtained from all participants prior to their inclusion in the study.

## Results

Patients experienced an average of 20 (15–25) headache attacks per month with an intensity of 7 (6–7) points on the VAS, and high scores on the HIT-6 – 53 (51–55). (Table 1). Sleep assessment scales identified sleep disturbances of varying severity. Most patients rated their sleep as “poor” across different metrics, which is confirmed by high scores on the PSQI - 12.12 [11.27–12.98], the Morin Insomnia Severity Scale - 14 (13–16), and the VAS for sleep – 6 (5–8) points.

**Table 1:** Comparison of pain scores in patients with mild to moderate insomnia.

Indicator	Morin insomnia severity index		p-Value
	Subthreshold insomnia	Moderate insomnia	
Frequency of headache days, Me [Q1; Q3]	18.00 [15.00; 25.00]	20.00 [17.00; 24.50]	0.393
Headache intensity (VAS), Me [Q1; Q3]	7.00 [7.00; 7.00]	7.00 [5.00; 7.25]	0.429
HIT-6, Me [Q1; Q3]	52.00 [51.00; 55.00]	53.50 [52.00; 55.00]	0.361
CSI, Me [Q1; Q3]	37.00 [34.00; 49.00]	53.00 [39.75; 56.50]	< 0.001^a^

^a^Statistically significant at p<0.05.

A statistically significant difference between the subjective perception of sleep and TST measured by polysomnography is noteworthy (340.59±59 and 513.47±52.26 min, respectively, p<0.001). 

**Figure 1:**
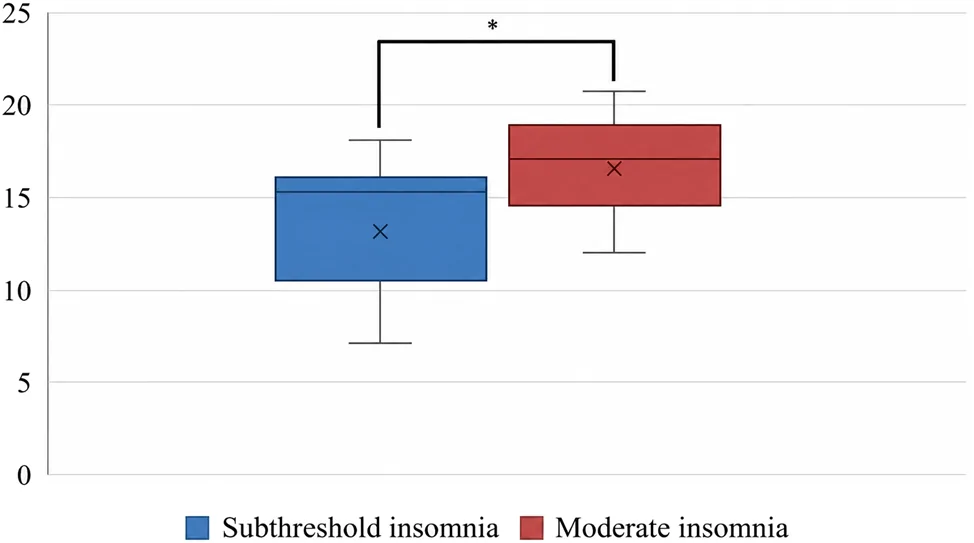
Beck depression inventory.

**Figure 2:**
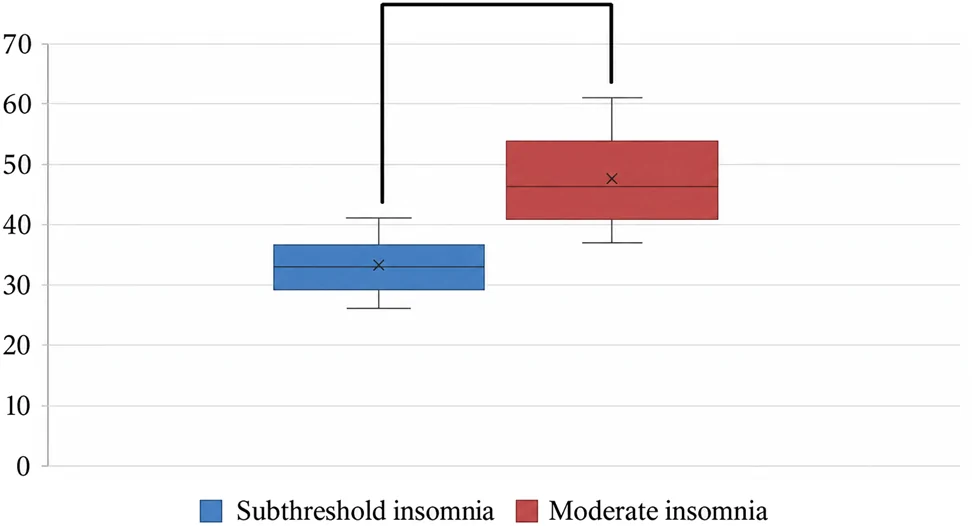
Sheehan anxiety scale.

Patients had comorbid depression - 14 (10–18) points on the BDI, and anxiety - 38 (26–51) points on the Sheehan Anxiety Scale (Figures 1 and 2).

A high degree of pain catastrophizing was noted (20.91 [17.56–24.56]), alongside reduced quality of life according to the SF-12 (30 [24–35]) and the Subjective Well-Being Scale (7 [5–9] points). Indirect signs of reduced quality of life included moderate to low scores on the VAS for health: 60 (50–70).

In accordance with the study aim, patients were divided into two groups based on insomnia severity (Morin Insomnia Severity Index): subthreshold insomnia (less than 14 points, n=33) and moderate-to-severe insomnia (15 points or more, n=32).

**Figure 3:**
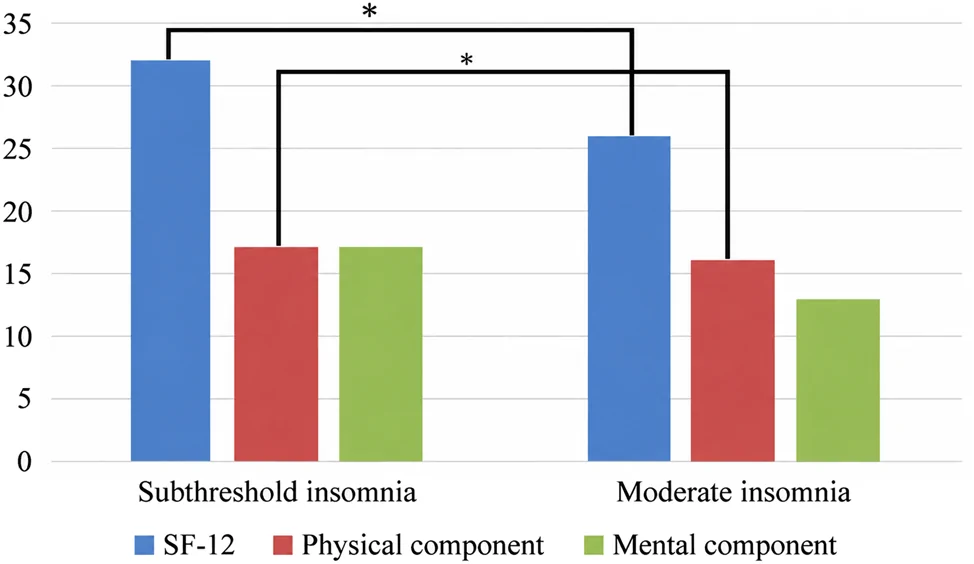
The short form health survey.

A comparative analysis of the groups showed that patients with more severe insomnia had statistically significant higher levels of anxiety (p<0.001) and depression (p=0.025) (Pic. 2) . These patients also rated their quality of life lower on the SF-12 (p=0.002) and the Subjective Well-Being Scale (p<0.019) (Figure 3). A notably higher level of central sensitization was observed in patients with more severe insomnia (p<0.001).

Correlation analysis in the mild insomnia group (Table 2) revealed the following: higher scores on the CSI were associated with increased wake time after sleep onset and reduced duration of REM sleep. An increase in depression levels correlated with a decrease in slow-wave sleep (SWS) duration and a reduction in S3 sleep stage duration. Increased anxiety was associated with greater wake time after sleep onset. Higher HIT-6 scores correlated with reduced total sleep time, lower sleep efficiency, shorter REM sleep duration, and increased wake time after sleep onset.

**Table 2:** Correlation analysis of polysomnographic and non-somnologicalindicators in the group with mild insomnia.

Indicator	Correlation characteristic		
	ρ	Strength of association (Chaddock scale)	p-Value
Sleep efficiency – HIT-6	−0.864	High	0.006
TST – HIT-6	−0.807	High	0.015
SWS – BDI	−0.77	High	0.041
REM sleep – CSI	−0.932	Very high	< 0.001
REM sleep – HIT-6	−0.888	High	0.003
Sleep stage 3 – BDI	−0.837	High	0.010
WASO – CSI	0.779	High	0.023
WASO – Sheehan anxiety scale	0.755	High	0.030
WASO – HIT-6	0.921	Very high	0.001

The following correlations were observed in the group with moderate-to-severe insomnia (Table 3). An increase in scores on the Subjective Well-Being Scale (indicating a worsening quality of life) was associated with a decrease in the duration of both S2 and S3 sleep stages. An increase in scores on the Pain Catastrophizing Scale, primarily driven by the “Rumination” subscale, correlated with a reduction in total sleep time.

**Table 3:** Correlation analysis of polysomnographic and non-somnological indicators in the group with severe insomnia.

Indicator	Correlation characteristic		
	ρ	Strength of association (Chaddock scale)	p-Value
TST - “Rumination” subscale	−0.746	High	0.021
REM sleep – Headache intensity, VAS	−0.766	High	0.016
Sleep stage 2 – Subjective well-being scale	−0.716	High	0.030
Sleep stage 2 – Sheehan anxiety scale	−0.712	High	0.031
Sleep stage 2 – SF-12	0.839	High	0.005
Sleep stage 2 - “Helplessness” subscale	−0.734	High	0.024
Sleep stage 3 – Subjective well-being scale	−0.743	High	0.022
Sleep stage 4 – Mental component SF-12	−0.745	High	0.021

An increase in anxiety levels measured by the Sheehan Anxiety Scale was associated with a decrease in the duration of S2 sleep. A decline in quality of life was also linked to a reduction in S2 sleep duration. Furthermore, a shorter duration of S2 sleep was correlated with higher scores on the “Helplessness” subscale of the Pain Catastrophizing Scale.

An increase in scores on the VAS for headache intensity was associated with a decrease in the duration of REM sleep. A reduction in the duration of the S4 component of slow-wave sleep was linked to lower scores on the “Mental Health” component of the SF-12.

## Discussion

Our study confirms the high prevalence of sleep disturbances in patients with CTTH, consistent with prior epidemiological and clinical observations [61], [62], [63], [64]. Insomnia was present in all participants, reflecting its frequent co-occurrence with CTTH and supporting evidence that sleep disturbances may represent a core component of the disorder. Due to the scarcity of CTTH patients with fully normal sleep, our comparison group included individuals with mild insomnia, while the main cohort comprised patients with moderate to severe insomnia, classified using the Morin Insomnia Scale. Emotional disturbances, including anxiety and depressive symptoms, were prominent in our cohort and appear to act as intermediaries between headache severity and sleep impairment, with bidirectional exacerbation [15], [16], [17], [18], [19]. Sleep disturbances can reduce daytime physical activity, potentially amplifying pain perception, while excessive time in bed attempting to mitigate pain often worsens sleep quality. Central sensitization mechanisms likely accelerate in patients with comorbid CTTH and insomnia, increasing both nociceptive sensitivity and the severity of sleep disturbances. This observation aligns with prior evidence linking heightened pain perception to insomnia severity, as well as hormonal and gender-specific cortical modulation of pain [20].

Our correlation analyses demonstrated strong associations between objective and subjective sleep parameters, emotional state, quality of life, and pain sensitivity. Notably, patients frequently underestimated their total sleep duration, potentially reflecting heightened nocturnal arousal and fragmented sleep perception caused by chronic pain. The severity of insomnia also correlated with a greater impact of headaches on quality of life, emphasizing the intertwined nature of sleep disruption, pain sensitization, and emotional dysfunction. Mechanistically, these clinical observations can be explained by overlapping peripheral and central pathways implicated in both CTTH and insomnia. Peripheral mechanisms in CTTH, including sustained pericranial muscle tension and myofascial trigger points, contribute to nociceptor sensitization and local ischemia [21], [22], [23], [24]. This peripheral input facilitates central sensitization through enhanced excitatory neurotransmission (glutamate, CGRP), activation of NMDA, mGluR, NK1, and purinergic receptors, and intracellular signaling cascades (PKA, PKC, CaMKII, ERKs) [25], [26], [27], [28], [29], [30], [31], [32]. Nitric oxide dysregulation and mitochondrial dysfunction further amplify neuronal excitability, promote oxidative stress, and activate inflammasomes (e.g., NLRP3), increasing pro-inflammatory cytokine production (IL-1β, IL-6, TNF-α) [33], [34], [35], [36], [37], [38], [39]. Coenzyme Q10 deficiency may exacerbate oxidative stress and impair neuronal energy homeostasis, further lowering the threshold for headache chronification [40]. Similarly, insomnia involves dysregulation of circadian and homeostatic mechanisms, oxidative stress, mitochondrial dysfunction, neuroinflammation, and neurotransmitter imbalance [41], [42], [43], [44], [45], [46], [47], [48], [49], [50], [51], [52], [53], [54], [55], [56], [57], [58], [59], [61]. Activation of microglia and astrocytes, excessive ROS/RNS production, peripheral immune activation, and impaired antioxidant responses (e.g., AMPK, NRF2, Prdx systems) contribute to neuronal hyperexcitability and disrupted sleep architecture. Sleep loss impairs mitochondrial respiration, decreases ATP production, and enhances oxidative stress, creating a feedback loop that reinforces both insomnia and pain sensitivity. Neurotransmitter dysregulation – including serotonergic, noradrenergic, dopaminergic, histaminergic, and cholinergic pathways – further destabilizes sleep-wake homeostasis and modulates pain perception [60], 65], 66].

The clinical manifestation of these shared mechanisms is a “vicious cycle,” whereby chronic pain, sleep disturbance, and affective dysregulation mutually reinforce one another. Consistent with our findings, previous studies report high rates of psychiatric comorbidity in CTTH, including generalized anxiety disorder (70.6 %), major depressive disorder (54.1 %), dysthymia, and panic disorder [67], [68], [69]. These data highlight the importance of evaluating both sleep and affective components when managing patients with CTTH. Taken together, our results and the mechanistic evidence support a model in which CTTH and insomnia arise from overlapping neurobiological, inflammatory, mitochondrial, and neurotransmitter-mediated pathways. Effective therapeutic strategies should therefore target not only pain but also sleep quality and emotional regulation to disrupt this reinforcing pathological cycle.

## Conclusions and future directions

The clinical and statistical association between insomnia, chronic tension-type headache (CTTH), and affective disturbances herein investigated underscores the presence of complex, interrelated pathogenetic mechanisms in this multimorbidity. Mechanisms such as neuroinflammation, oxidant-antioxidant imbalance, mitochondrial dysfunction, hormetic disregulation, and impairment of neurotransmitter systems are involved and interwined. Consistently, elucidating the causal relationships within this network of interactions could have substantial implications for the prevention and management of chronic headache and associated sleep disorders. A mechanistic understanding of these shared pathways expands therapeutic options, including interventions aimed at correcting mitochondrial dysfunction, supporting redox homeostasis, and restoring circadian regulation. These targeted strategies hold promise for improving outcomes in patients with CTTH comorbid with insomnia and merit further investigation. Our study confirms the frequent co-occurrence of chronic tension-type headache, insomnia, and affective disorders, reflecting the interplay of multiple pathophysiological factors, thereby suggesting a bidirectional, self-reinforcing “vicious cycle” in which pain, sleep disturbance, and emotional dysregulation exacerbate one another, resulting in impaired daily functioning and reduced adaptive capacity. Insomnia independently contributes to heightened pain sensitivity, amplifying the negative impact of CTTH on quality of life. The strongest associations were observed in patients with severe insomnia, suggesting that sleep disturbances compromise endogenous pain modulation and resilience. These observations support a multimodal therapeutic approach, combining sleep normalization with psychosocial interventions and nutritional support. Optimizing sleep quality in patients with chronic tension headache not only alleviates insomnia but also enhances pain coping, emotional well-being, and overall life satisfaction.
